# Contributions and complexities from the use of *in vivo* animal models to improve understanding of human neuroimaging signals

**DOI:** 10.3389/fnins.2014.00211

**Published:** 2014-08-19

**Authors:** Chris Martin

**Affiliations:** Department of Psychology, The University of SheffieldSheffield, UK

**Keywords:** neurovascular, functional magnetic resonance imaging, rodent, neuroimaging, hemodynamic

## Abstract

Many of the major advances in our understanding of how functional brain imaging signals relate to neuronal activity over the previous two decades have arisen from physiological research studies involving experimental animal models. This approach has been successful partly because it provides opportunities to measure both the hemodynamic changes that underpin many human functional brain imaging techniques and the neuronal activity about which we wish to make inferences. Although research into the coupling of neuronal and hemodynamic responses using animal models has provided a general validation of the correspondence of neuroimaging signals to specific types of neuronal activity, it is also highlighting the key complexities and uncertainties in estimating neural signals from hemodynamic markers. This review will detail how research in animal models is contributing to our rapidly evolving understanding of what human neuroimaging techniques tell us about neuronal activity. It will highlight emerging issues in the interpretation of neuroimaging data that arise from *in vivo* research studies, for example spatial and temporal constraints to neuroimaging signal interpretation, or the effects of disease and modulatory neurotransmitters upon neurovascular coupling. We will also give critical consideration to the limitations and possible complexities of translating data acquired in the typical animals models used in this area to the arena of human fMRI. These include the commonplace use of anesthesia in animal research studies and the fact that many neuropsychological questions that are being actively explored in humans have limited homologs within current animal models for neuroimaging research. Finally we will highlighting approaches, both in experimental animals models (e.g. imaging in conscious, behaving animals) and human studies (e.g. combined fMRI-EEG), that mitigate against these challenges.

## Introduction

“One of the difficulties in understanding the brain is that it is like nothing so much as a lump of porridge.”

Richard L. Gregory (Gregory, 1997)

Human functional brain imaging techniques now play a prominent role in much neuroscience and psychological research. Unlike many other organs in the body, assigning function or aspects of functions, in space or time to particular components of the brain based on even a very detailed analysis of its structure alone is extremely difficult. Although we have learned much from neuropsychological research in human subjects or experimental animal research studies, there can be little doubt that the possibilities for understanding afforded by any technique that can provide a spatiotemporal readout of changes in brain function are immense. It is thus perhaps unsurprising that the rate of publication of scientific papers incorporating functional neuroimaging now exceeds 10 per day (Kim and Ogawa, 2012). Beyond academic communities, these tools and the images they produce have also captured public interest and provided renewed opportunities for neuroscientists to engage with publics on both scientific matters and upon issues at the science-society interface (Racine et al., 2005).

**Figure 1:**
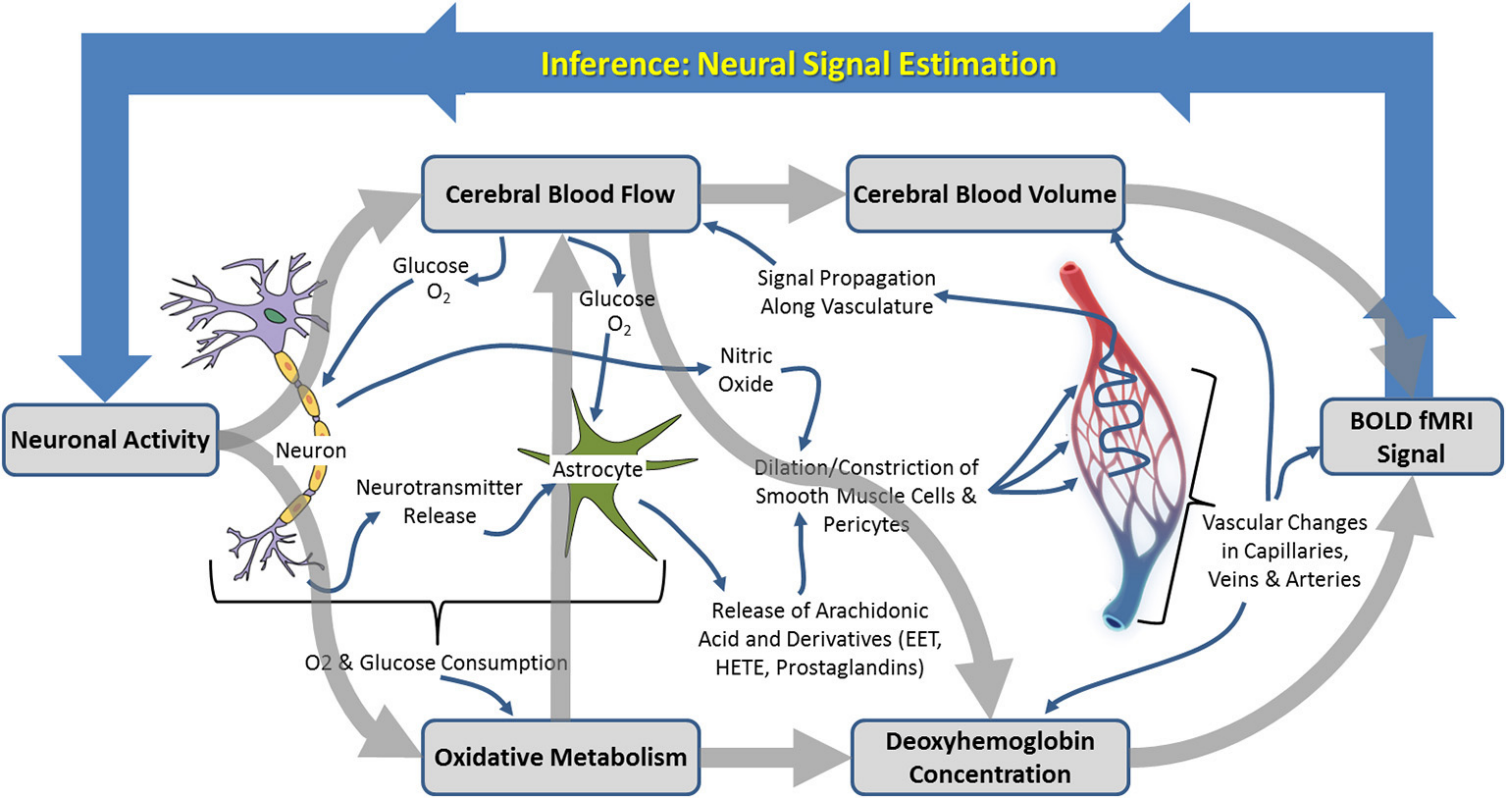
**Schematic illustration of the neurophysiological processes underpinning hemodynamic neuroimaging signals**. The boxed processes linked by thick gray arrows around the outside represent components of interest to those focussing on “parametric neurovascular coupling,” whereas the more detailed processes illustrated in the center represent important concepts in for investigation of “physiological neurovascular coupling.” The relationships between the illustrated biophysical or physiological components, as well as the baseline conditions upon which changes are superimposed, may be mediated by the factors illustrated in **Figure 6**.

For more than 20 years, the non-invasive neuroimaging technique of functional magnetic resonance imaging (fMRI) based on blood oxygen level dependent (BOLD) signal changes has been used to estimate neural signals in the human brain (Ogawa et al., 1990). Prior to this, positron emission tomography (PET) firmly established the possibilities for human neuroimaging based on surrogate hemodynamic markers. Since the human application of these hemodynamic based neuroimaging techniques, substantial effort has been directed toward improving our understanding of functional brain imaging signals, the hemodynamic changes that give rise to them, and the relationship of these changes to underlying neuronal activity (Figure 1). Although it is widely appreciated amongst scientific communities that the relationships between activity within heterogeneous neuronal populations and neuroimaging signals are complex, rather indirect, and incompletely understood, many research papers continue to report neuroimaging signals as “*measures of neuronal activity*,” which they are not. In addition, it has been shown that the presence of brain “activation” images in scientific papers results in higher ratings of scientific quality even where the images provide no additional information to the reader (Mccabe and Castel, 2008). It is thus increasingly important that we both build our understanding of precisely what functional neuroimaging signals do tell us about brain activity, and ensure that improved frameworks for the interpretation of brain imaging data are effectively communicated. In this review, we will use the terms neuroimaging or functional brain imaging to refer to hemodynamic imaging techniques as applied to human subjects, rather than the broader definition which includes electroencephalography (EEG) or magnetoencephalography (MEG).

Neuroimaging signals arise because of a coupling between changes in neural activity, metabolism, and hemodynamics (blood flow, oxygenation, and volume) in the brain, termed neurovascular coupling (Figure 1; Villringer, 1997; Logothetis and Wandell, 2004; Logothetis, 2008). Studies of neurovascular and neurometabolic coupling have therefore been central to the progress that has been made so far in investigating what neuroimaging signals tell us about neuronal activity. Important insights have arisen from research using animal models, in our laboratory and elsewhere, in which detailed measurements of functional brain imaging signals and/or the hemodynamic and neuronal events underpinning them can be made (Mathiesen et al., 2000; Norup Nielsen and Lauritzen, 2001; Smith et al., 2002; Devor et al., 2003, 2005; Martindale et al., 2003; Jones et al., 2004; Sheth et al., 2004; Berwick et al., 2005, 2008; Hewson-Stoate et al., 2005; Martin et al., 2006a,b, 2012; Hillman et al., 2007; Franceschini et al., 2008; Boorman et al., 2010; Kim et al., 2010). It is possible, in animal models, to explore in detail the changes that occur in each component of the complex system linking neuronal activity to neuroimaging signal changes, as illustrated in Figure 1. Thus, a number of landmark papers (e.g. Logothetis et al., 2001) have now established the general validity of fMRI signal changes as indicators of altered neuronal activity: there is overwhelming empirical evidence that increases in BOLD fMRI signals in healthy cortical structures reflect increased neuronal activity in those structures. However, the boundaries of this broad statement and limits to its generalizability are becoming increasingly important as neuroimaging is applied to study the whole brain in both health and disease contexts.

The focus of this paper will therefore be upon the exceptions, complexities and remaining uncertainties around neuroimaging signal interpretation, as revealed predominantly by *in vivo* experimental animal research into neuroimaging signals and neurovascular coupling. We shall begin by outlining the main approaches to investigating neuroimaging signals and neurovascular coupling in experimental animal models, including a brief overview of the major techniques. We shall then review some of the key research questions that these approaches allow us to address along with some of the main insights provided thus far. We shall review recent research highlighting areas where the relationships between neuronal activity and hemodynamic changes are more complex and discuss the implications of this for neural signal estimation in human neuroimaging. Finally we will turn attention to the experimental animal models themselves, their limitations, as well as new possibilities to investigate neurovascular coupling directly in human subjects.

## Investigating neuroimaging signals using experimental animal models

**Figure 2:**
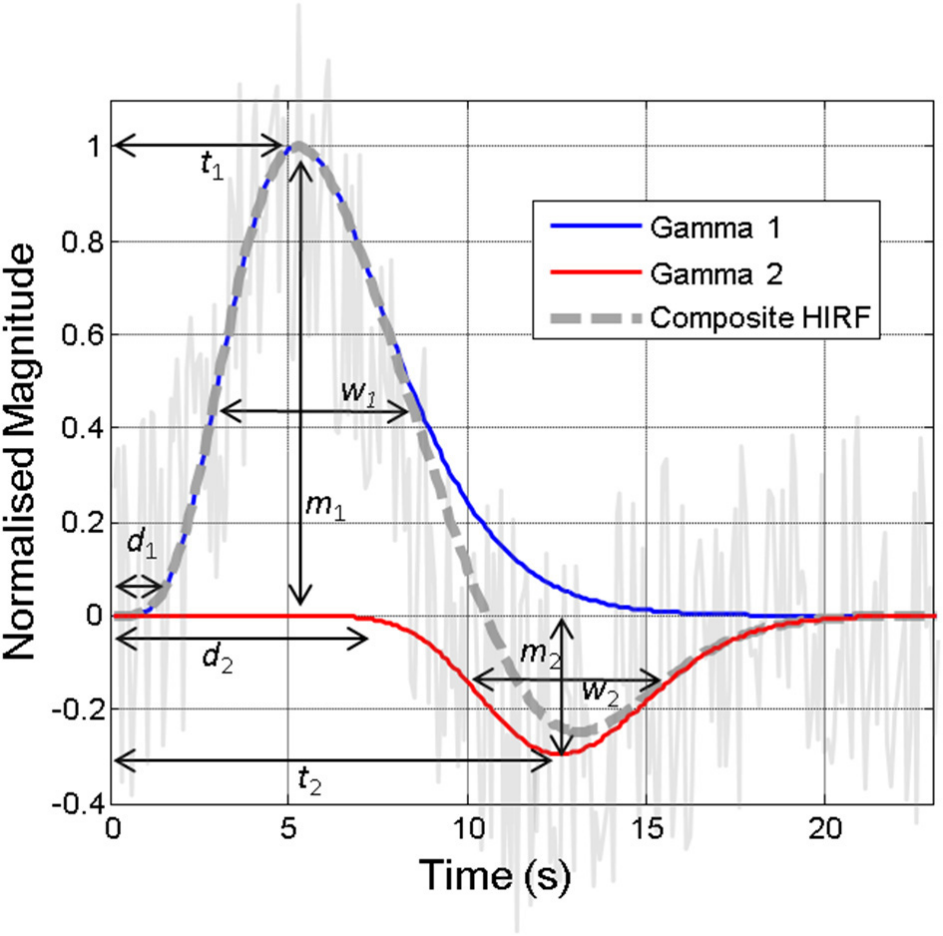
**Illustration of the hemodynamic impulse response function (HIRF, dashed gray line), which typically comprises two gamma functions (red and blue lines), together approximating the hemodynamic responses present in often noisy acquired neuroimaging data (solid gray line)**. The various parameters required to specify the HIRF are illustrated. Variations in these parameters that are not accounted for can lead to inaccurate detection of “activity” in neuroimaging studies. Some of the factors that might alter these parameters are illustrated in **Figure 6**.

Research using experimental animal models to investigate neuroimaging signals and their relationship to neuronal activity has approached the issue from two converging perspectives. On the one hand there has been an emphasis on determining the parametric relationships between neuronal activity and neuroimaging signals, including characterization of the mathematical relationships between signals, estimation of the hemodynamic impulse response function (HIRF, see Figure 2 and section Characterization of hemodynamic impulse response functions), and the development of comprehensive biophysical models of neurovascular coupling. We shall refer to this approach as being concerned with “parametric neurovascular coupling,” emphasizing the concurrent measurement of signals in different components of the neurovascular-neuroimaging system, illustrated schematically in Figure 1 (outer processes). On the other hand, attempts have been made to determine the physiological mechanisms of neurovascular coupling, with a focus on the key chemical mediators and modulators, and the relative involvement of different cell types including astrocytes, neurons, pericytes, endothelial cells, and vascular smooth muscle cells. We shall refer to this approach as being concerned with “physiological neurovascular coupling,” emphasizing the biochemical and physiological mechanisms that mediate the relationships between the neuronal, metabolic, and hemodynamic components, illustrated in Figure 1 (inner detail). The former approach is more directly relevant to the development of strategies to analyze neuroimaging data and our ability to make general judgments regarding the effects of experimental manipulations on the magnitude of underlying neuronal signals, whereas the latter is critical if we are to improve our detailed understanding of the neurophysiological events that these signals represent. Convergence of both perspectives is important for progressing our ability to estimate neural signals from functional brain imaging data acquired in an increasingly broad range of scenarios. With this distinction in mind, we shall now briefly walk through the principle methodologies being applied in this field and outline how they contribute to either approach. A summary of the main techniques used to improve understanding of neuroimaging signals in experimental animal models is provided in Table 1 and for a fuller review the reader is directed to recent publications by Devor et al. (2012, 2014) and Zhao et al. (2014c).

**Table 1:** **Overview of principle methods used to investigate neuroimaging signals and neurovascular coupling**.

**Technique**	**Data streams**	**Main advantages**	**Relative disadvantages**	**Usable in humans?**
High field fMRI	BOLD, CBV, CBF	Cross-species technique, whole brain imaging	High cost, difficult to combine with other techniques, limited spatial and temporal resolution	Yes
Near infrared spectroscopy	Oxy-, deoxy, and total hemoglobin	High temporal resolution, low cost human neuroimaging	Poor spatial resolution, limited depth penetration	Yes
Diffuse optical tomography	Oxy-, deoxy, and total hemoglobin	Cross-species technique	Poor spatial resolution, limited depth penetration	Yes
Optical imaging spectroscopy	Oxy-, deoxy, and total hemoglobin	Combination of good temporal and spatial resolution, easy to combine with other techniques	Limited depth penetration, cortical surface only	Intraoperative only
Photoacoustic tomography	Oxy-, deoxy, and total hemoglobin, microcirculation	Whole brain imaging, high spatial resolution	Relatively low temporal resolution	No
Optical coherence tomography	Blood flow (microcirculation)	Absolute measurement of flow, good depth penetration and spatial resolution	Only cortical (surface structure) imaging possible	No
Laser speckle contrast imaging	Blood flow (2D)	High sensitivity, can image through skull	Limited depth penetration, spatial resolution lower then optical techniques	No
Confocal microscopy	Blood flow, tissue oxygen, microcirculation, cellular activity	Excellent spatial and temporal resolution, can measure neuronal and vascular markers	Loss of spatial resolution at depth, higher risk of photobleaching	No
Two-photon microscopy	Blood flow, tissue oxygen, microcirculation, cellular activity	Highest spatial and temporal resolution, neuronal and vascular markers, good depth penetration	Relatively costly, not easy to combine with other techniques	No
Voltage sensitive dye imaging	Cellular activity	High spatial and temporal resolution measurement of cellular activity	Difficult to combine with other optical readouts, risk of toxicity from dyes	No
Laser doppler flowmetry	Blood flow (point mesaurement)	Easy to combine with other techniqies	Point measurement only	No
Tissue oxygen voltammetry	Tissue oxygen (point measurement)	High temporal resolution	Point measurement only, limited spatial precision	No
Tissue oxygen polarography	Tissue oxygen (point measurement)	Very high resolution recording	Point measurement only, fragile electrodes	No
Tissue oxygen luminescence	Tissue oxygen (point measurement)	Easy to use, good temporal resolution	Point measurement only, limited spatial precision	No
Invasive electrophysiology (Various)	Single or multi-unit activity, local field potentials	Highly localised recording, optimal temporal resolution	limited compatibility with fMRI, risk of damage to brain tissue from electrode	No
Non-invasive electrophysiology (EEG, MEG)	Event-related potentials, current sources and sinks	High temporal resolution, low cost human neuroimaging	Limited spatial resolution	Yes

### fMRI for interrogating neuroimaging signals

To some extent it is “by definition” that investigations of neurovascular coupling usually require measurement of both the neural and vascular components of the overall system. In parametric neurovascular coupling research this is predominantly the case and so experimental designs in which multiple data acquisition methods can be combined have become the workhorse of this field. Because of the high degree of inter-trial (and inter-subject) variability inherent in hemodynamic measurements in particular, concurrent acquisition of neuronal and hemodynamic data provides significant statistical and data modeling advantages (in addition to reducing animal numbers). Combining neuroimaging techniques that are used in humans (such as fMRI) with electrophysiological techniques to measure neuronal signals would appear the most parsimonious approach to elucidating neuroimaging-neural signal relationships. These data provide direct insight into the quantitative relationships between neuroimaging and neural signals. Furthermore, small-animal fMRI systems, though a combination of reduced bore size (and accordingly other hardware including gradient coils), smaller radiofrequency coils and increased field strength are routinely able to resolve voxels approximately an order of magnitude smaller than obtainable in human fMRI. High field strength fMRI in animal models has enabled researchers to study the fine detail of hemodynamic responses, with sub-second temporal precision and in-plane voxel sizes of <100 μm (see section Heterogeneous distribution of fMRI signals sources revealed by high field strength fMRI). However, combining this approach with direct measures of neuronal activity is problematic for a number of reasons. Firstly, electrodes to record neuronal activity will introduce artifacts into the imaging data. These can be minimized by careful electrode design, choice of materials, and image acquisition parameters, but it is very difficult to completely avoid distortion of imaging data at precisely the point where it is of most interest (where the electrode is positioned). Secondly, fMRI in particular makes use of large, rapidly changing electromagnetic fields as part of the data acquisition process which can severely distort the electrophysiological measurement of neural signals. Whilst an increasing number of laboratories have been able to find ways around this (e.g. Huttunen et al., 2008), it is technically challenging and often requires substantial “development time” on an MRI system, making it a costly process. Thirdly, there are also limits to temporal and spatial resolution of PET and to a lesser extent fMRI. Although high-field strength fMRI is now able to achieve impressive results, as discussed further below, convergence upon the spatiotemporal scales at which neuronal activity operates or blood flow is directly regulated, remains elusive. Finally, a major barrier to the use of non-invasive imaging techniques for research in this field is cost: preclinical MRI facilities (for rats/mice) may charge >$3000 per day, whereas facility costs are typically an order of magnitude lower for using many of the alternate *in vivo* approaches as summarized in Table 1.

### Optical methods for improved resolution and multimodal data acquisition

**Figure 3:**
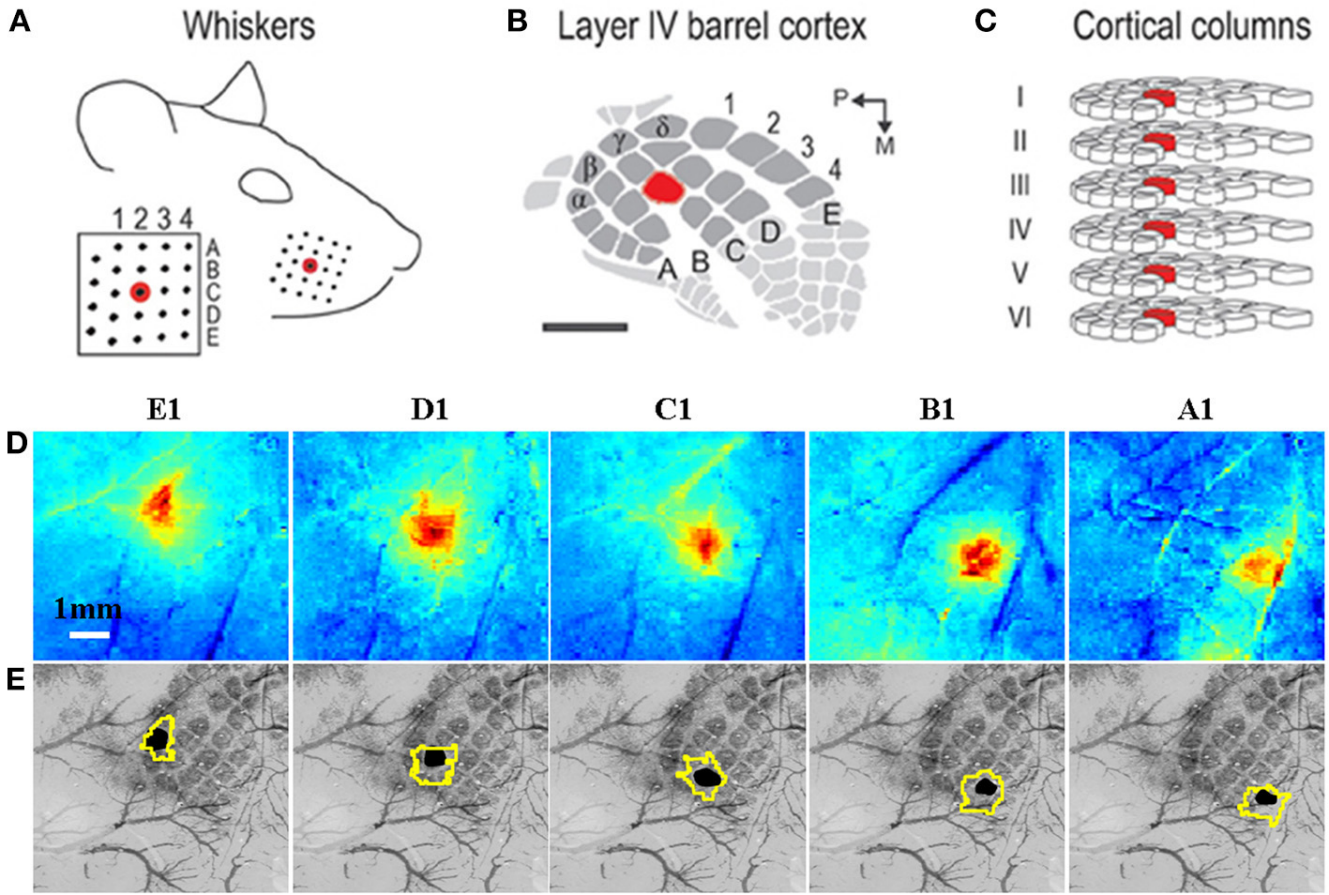
**Grid-like arrangement of the rodent facial whiskers (A) is topographically preserved with mapping in somatosensory cortex (B) of individual whiskers to individual cortical columns (C)**. **(A–C)** adapted from Chen-Bee et al. (2012). **(D)** Measurement of total hemoglobin concentration changes during stimulation of individual whiskers (A1–E1) produces spatiotemporal activation maps that allow spatial discrimination of activation primarily located in each corresponding cortical column. **(E)** Co-registered surface (vasculature) and cortical histological sections with the stimulated barrel highlighted in black to verify anatomical specificity. The contour around each stimulated barrel is the activated total hemoglobin region defined as all pixels with 50% of the peak response from a mean image of the last 4 s of the 16 s stimulation period. **(D,E)** adapted with permission from Berwick et al. (2008).

Because of these limitations, and because more invasive approaches can be used in experimental animals models, methods of making hemodynamic measurements that require more direct brain access have become well established. An increasingly wide range of techniques make use of the light absorption and/or scattering properties of brain tissue, and specifically the hemoglobin present in the vascular system, in order to obtain high spatial and temporal resolution readouts of hemodynamic changes. These techniques usually require visualization of the brain either through a craniotomy or a thin cranial window (the skull is thinned to translucency over the imaged brain tissue). One popular technique, intrinsic signal optical imaging (Malonek and Grinvald, 1996), measures changes in the concentration of oxy- and deoxy-hemoglobin and is able to resolve changes at the level of 10 s of microns and 10 s of milliseconds (Figure 3). This technique is particularly amenable to combination with other methods, such as the implantation of microelectrodes to simultaneously record neuronal activity, or the addition of other probes to record blood flow changes or tissue oxygenation. Another advantage of this approach is that the cost and complexity of the imaging system is much reduced compared to MRI systems. However, a major disadvantage of many standard optical imaging approaches is the limited depth penetration due to light scattering and absorption by tissue: systems exploiting light in the visible wavelength range are limited in signal acquisition to just the first few hundred microns of cortical tissue. The related techniques of near infrared spectroscopy which uses light at longer wavelengths to penetrate several centimeters of tissue, or diffuse optical tomography which uses an EEG-style array of detectors (and sources), have the advantage that they are used in both human and animal studies (they can penetrate the skull), but there is a severe trade off in terms of spatial resolution due to light scattering effects. This class of methodologies provides information relevant to both parametric and physiological neurovascular coupling approaches (although typically individual research studies will emphasize one other the other approach). This is because fairly detailed measurement of different aspects of the hemodynamic and neuronal responses can be combined with pharmacological manipulations to enable interrogation of relevant biochemical pathways (e.g. see reviews by Carmignoto and Gomez-Gonzalo, 2010; Cauli and Hamel, 2010).

### Microscopy methods for cellular level resolution and research into mechanisms

Two-photon laser scanning microscopy (2PSLM) in an increasingly popular technique that is able to record both neuronal and hemodynamic events at cellular level resolution (see Shih et al., 2012a, for an excellent recent review) thus enabling physiological neurovascular coupling to be investigated. Fluorescent reporter molecules are used to provide information about intracellular events, vascular responses, blood flow and other phenomena and these can be measured at extremely high spatial resolution due to the very narrow focal plane of 2PLSM. Additionally, the use of laser light with wavelengths in the near infrared range is able to achieve better tissue penetration than standard optical imaging techniques (up to 1 mm). It is also possible to “stimulate” neurophysiological events, for instance using calcium uncaging by photolysis (e.g. Takano et al., 2006), thus providing opportunities for very fine levels of control and recording of neurovascular function. This microscopic approach is often combined with highly specific pharmacological or electrophysiological manipulations which may be applied to individual cells. It can be performed *in vivo* or *in vitro*, and increasingly research groups will deploy the technique in both modes to address a specific question in order to combine the enhanced control, manipulation and data quality advantages of the *in vitro* approach with the demonstration of functional relevance that is best achieved *in vivo*. 2PLSM is therefore becoming established as a “gold standard” method for addressing physiological neurovascular coupling research questions. Disadvantages of 2PLSM include the requirement for the lens objective to be very close to the imaged tissue sample, making the combination of 2PLSM with other recording methods technically challenging. This could be a problem for relating the microscopic findings from 2PLSM to the more macroscopic vascular and neuronal events that neuroimaging measures or makes inferences about, respectively, and which is typically a strength of parametric neurovascular coupling approaches.

### Monitoring neuronal activity

A major advantage of using animal models in this context is that it is possible to combine methods that measure hemodynamic changes (including non-invasive imaging tools such as small animal MRI) with other, usually invasive methods that can measure the changes in neuronal activity that underlie the hemodynamic events. For this latter purpose, electrodes can be implanted directly into the brain to record the activity of neurons and in some cases multi-site electrode probes are used which can capture activity at multiple locations in the brain or along the length of a functional unit such as the cortical column (Berwick et al., 2008). From these recordings it is possible to resolve both local field potentials (LFPs) (an aggregate measure of excitatory and to a lesser extent inhibitory synaptic, activity in a local cell population, primarily reflecting input) and spiking activity (action potentials, primarily reflecting output activity). Other, optical approaches, are able to produce high resolution 2 or 3-D maps of cellular activity (Akkin et al., 2010), often in combination with reporter dyes (Devor et al., 2007). Although these invasive approaches provide measurements of neuronal activity that share signal sources with non-invasive electrophysiological techniques that are used in human subjects, such as EEG or MEG, because of large differences in the spatial resolutions achievable and other factors, direct comparisons are not straightforward. In this respect, further research is needed to improve understanding of the relationships between the different measures of neuronal activity made in humans and animal models to underpin translation of findings between studies (e.g. Buzsáki et al., 2012).

**Figure 4:**
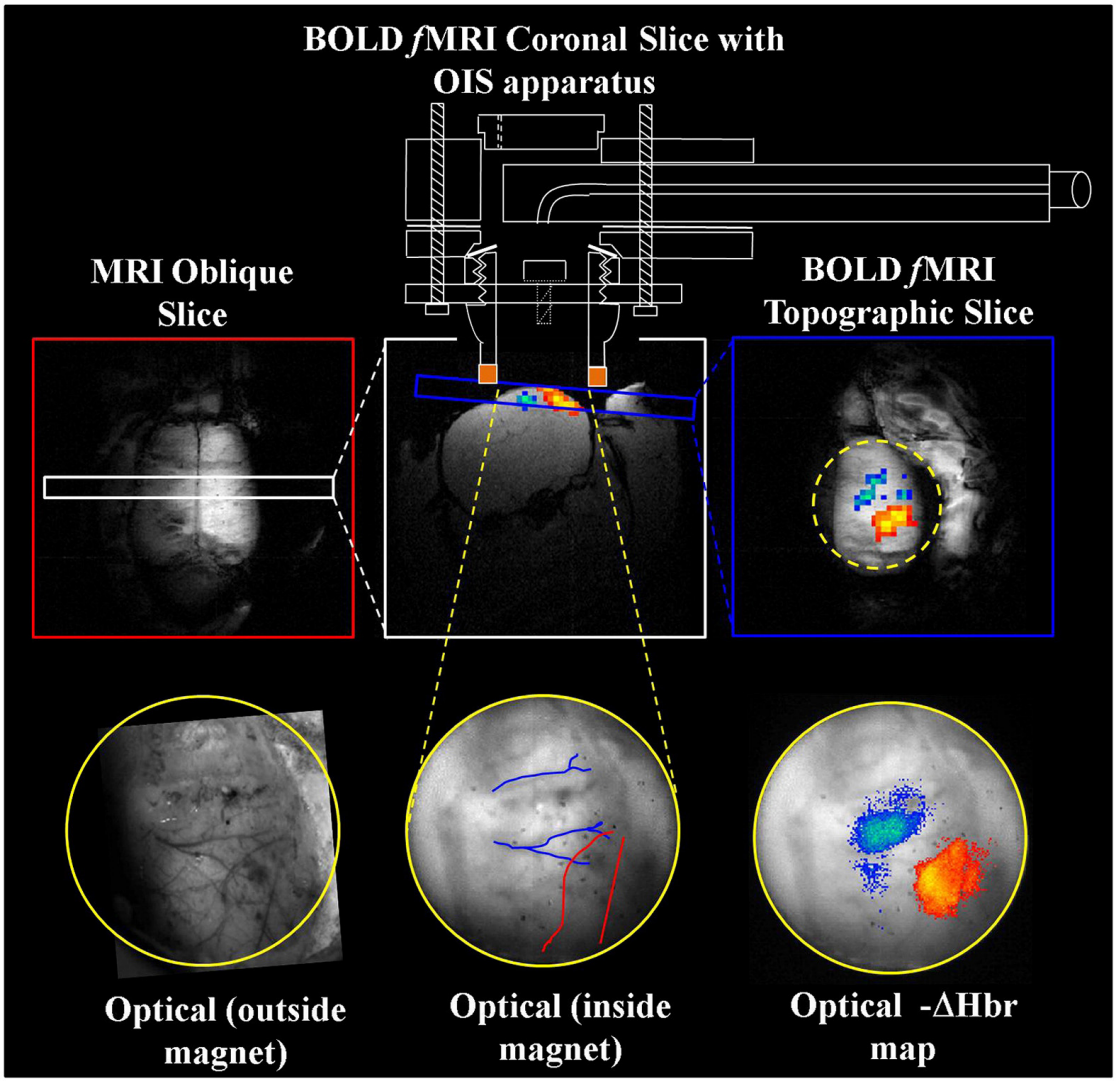
**Concurrent fMRI and optical imaging spectroscopy**. An oblique slice covering the dorsal surface of the brain (top left) is first used to identify a coronal (top center) or topographic slice (top right) containing the whisker barrel cortex for fMRI data acquisition. Apparatus allowing concurrent optical imaging is illustrated (top), consisting of a specially adapted MRI-compatible endoscope to transmit light to and from the brain surface. Optical imaging data is shown in the bottom panels, including the raw gray scale imaging of the cortical surface visualized through a thin cranial window (left and center), and activation induced changes in deoxyhemoglobin concentration (right) that correspond well to the concurrently acquired fMRI data (top right). Adapted with permission from Kennerley et al. (2012).

**Figure 5:**
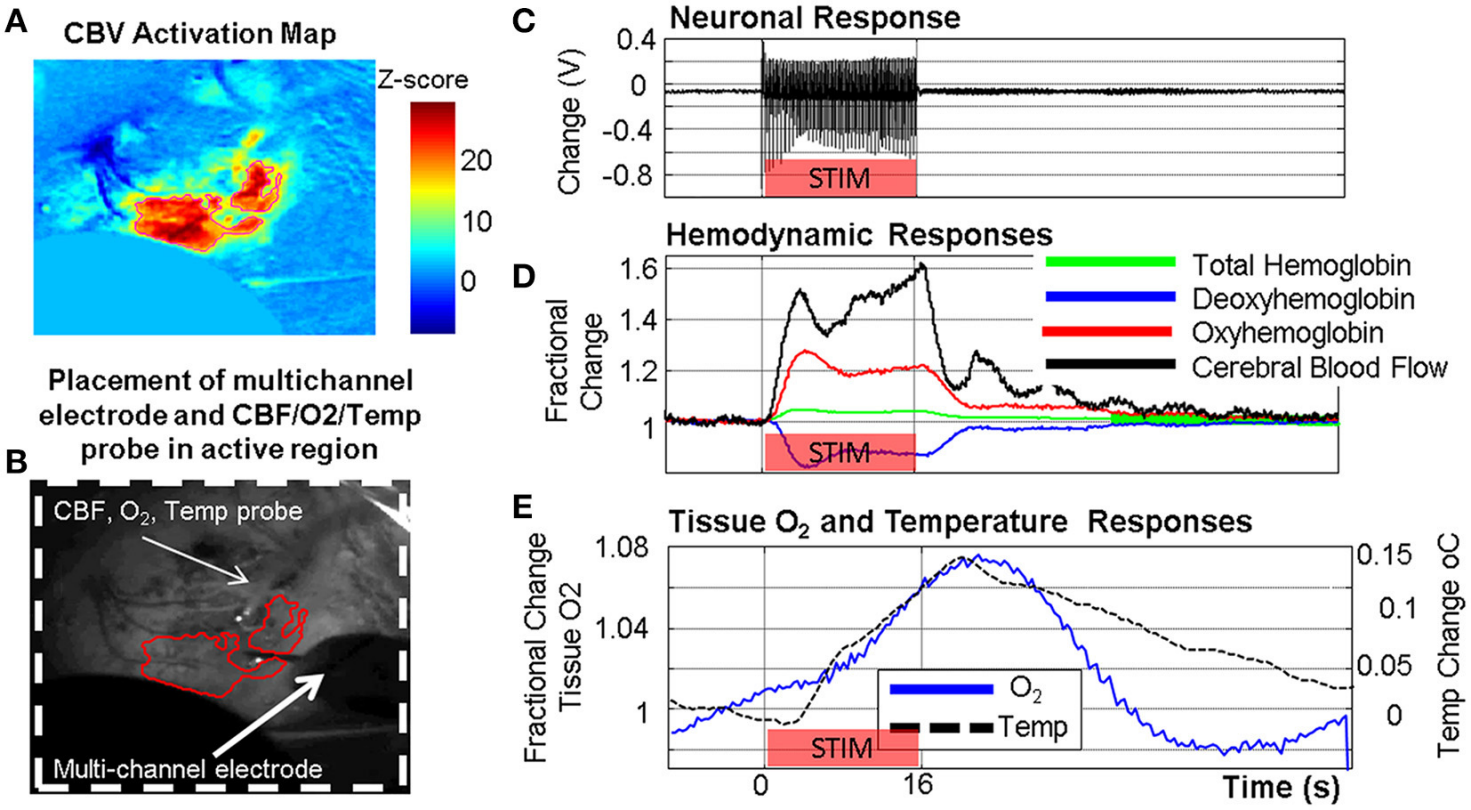
**Example of concurrent multi-modal measurement of neurovascular function developed in our laboratory showing simultaneously acquired neuronal, metabolic and vascular responses in the somatosensory cortex following a 16 s stimulation of the contralateral whisker pad**. **(A)** Cerebral blood volume (CBV) activation map from optical imaging spectroscopy (OIS). **(B)** Grayscale image of thin cranial window with measurement probes indicated. **(C)** Neuronal response (LFP shown). **(D)** Oxy/Deoxy/Total Hemoglobin changes from OIS with cerebral blood flow (CBF) changes from laser Doppler flowmetry. **(E)** Tissue oxygen and temperature responses.

The direct brain access, experimental control and stability afforded by the use of experimental animal models has enabled many combinations of the techniques or approaches described above and in Table 1 to be used in order to directly interrogate neuroimaging signals and neurovascular coupling (see Zhao et al., 2014c). For example, in our own laboratory we have established concurrent optical imaging and high field fMRI in rodents in order to provide insights into the hemodynamic constituents of BOLD fMRI signal changes (Figure 4). Working outside of the MRI environment we have been able to combined a wider range of measurement techniques for concurrent recording of various neuronal, metabolic, and hemodynamic signals in rodent somatosensory cortex (Figure 5), providing a multimodal readout of neurovascular function. The multimodal measurement techniques, when combined with other experimental tools such as transgenic animals or pharmacological manipulations, can provide new insights which bridge the “parametric” and “physiological” neurovascular coupling research approaches. This is especially the case when the resultant data is made available for the development of biophysical models which attempt to capture the mathematical relationships between important biological parameters (e.g. Zheng et al., 2010; Brodersen et al., 2011; Rosa et al., 2011).

### Possibilities for acute and reliable longer term data acquisition

Acute experiments involving animal models might be conducted over many hours, during which time baseline physiological parameters are carefully monitored and maintained within a narrow range. This enhanced window for data acquisition enables a more intensive exploration of the effects of manipulating independent variables (such as stimulation intensity, duration, frequency, repetition rate, multiple stimulus types) upon measured responses than would be possible in human subjects. Within such a time window it also possible to explore the effects of pharmacological or other manipulations on a within-subjects basis, obtaining neurovascular coupling readouts pre-, during-, and post-treatment. Because animals can be fixed with respect to the imaging apparatus more rigidly and for longer periods than would be possible in human subjects, test-retest reliability is very high. Chronic experimental designs are also possible where data from individual subjects can be acquired repeatedly over many weeks or months (Weber et al., 2006; Silva et al., 2011; Brydges et al., 2013; Martin et al., 2013). Although such approaches are also used in human neuroimaging studies, the high degree of experimental control possible in animal studies is particularly advantageous in light of the many factors which in humans can alter neurovascular coupling (e.g. as discussed elsewhere in this paper).

### Whole brain access

Human neuroimaging studies frequently report on responses to stimuli occurring across the brain, in both cortical and subcortical regions. In animal models it is possible to elicit controlled changes in neuronal activity in specific structures, including deep brain structures, which are not easily accessible to non-invasive stimulation techniques in order to refine our understanding of neurovascular coupling across the whole brain. In addition, as many brain structures are in receipt of multiple, convergent inputs that may or may not involve different cell types or neurotransmitters, it is possible, by independently stimulating convergent pathways, to improve our understanding what the composite neuroimaging signal is revealing about input patterns (e.g. see Enager et al., 2009; Krautwald and Angenstein, 2012; Krautwald et al., 2013).

## Key areas of insight from *in vivo* experimental animal research studies

In the following sections, we focus on four major themes that emerge from state-of-the-art research studies using animal models which target key questions in relation to understanding neuroimaging signals.

### Heterogeneous distribution of fMRI signals sources revealed by high field strength fMRI

Research carried out on high field strength, preclinical MRI systems has provided important insights into the hemodynamic composition of neuroimaging signals as well as the localization of separable signal components to specific vascular or neuronal architectures across the cortical laminae. This is a question not just relating to anatomical localization, but given our extensive understanding of how neural computation is distributed within the cortical columnar structure, goes to the core of precisely “what” fMRI signals reveal about neuronal activity in the context of cortical neuroimaging.

Recent studies in rodents and non-human primates have enabled the cortical hemodynamic response to sensory stimulation be elucidated as a function of cortical depth. Goense et al. (2012) studied both positive and negative BOLD signals, in addition to the commensurate CBV and CBF changes, in the primary visual cortex of anesthetized macaques. Although positive BOLD signals were associated with increases in both CBV and CBF, the depth profile varied for the BOLD, CBV, and CBF changes. Whilst BOLD increases were maximal at the cortical surface, CBF increases were maximal at approximately layer IV whilst CBV increases occurred with relative uniformity throughout the cortical layers. More intriguingly, negative BOLD signals occurred in regions where CBV increased and CBF decreased. Maximal negative BOLD responses occurred across the middle cortical layers whereas CBV responses were largest around layer IV and CBF responses were largest at the surface. This pattern of results suggest that the hemodynamic response to changes in neuronal activity that is measured in most neuroimaging studies (which lack the ability to resolve cortical layers) is an aggregate of a complex set of blood volume, flow and oxygenation response functions which appear to be heavily dependent upon an interaction of vascular and neuronal architecture. Goense et al., further conclude that these data provide evidence for differing neurovascular coupling mechanism operating at different cortical layers and underlying positive and negative BOLD signal changes. These data are in broad agreement with Shih et al. (2013) who used CBV-weighted fMRI at 11.7T in anaesthetized rats to resolve hemodynamic changes at a resolution that revealed cortical columnar like structure. CBV changes were again found to be maximal at ~layer IV. In this study, the laminar profile of neuronal activity was also measured (in a separate group of animals) and this revealed a mismatch between hemodynamic and neuronal activation-depth profiles. A further challenge to the specificity of BOLD fMRI signals to neuronal activity at the level of cortical laminae was reported by Herman et al. (2013), who calculated the laminar profile of changes in oxidative metabolism (CMRO_2_) in response to somatosensory stimulation in anesthetized rodents using a multimodal “calibrated fMRI” approach. Here, BOLD and CBV changes appeared spatially uncoupled to separately measured neuronal activity whereas CBF and CMRO_2_ changes were relatively well coupled to LFPs and multi-unit activity (spiking) respectively.

On the one hand the data obtained using these methods are problematic for fMRI: the neuronal activity mapping potential of BOLD signals in particular appears to be confounded by the neurovascular macrostructure of the cortical column (the spatial relationship of micro- and macro-vasculature to the neuronal and neurometabolic activity “sinks”). On the other hand, these data represent a new set of constraints that researchers can build into mathematical modeling and data analysis tools in order to improve the accuracy of inferences about neuronal activity. In addition, the increasing availability of higher field strength systems for research use and a wider use of multimodal fMRI acquisition approaches (i.e., acquiring CBF and/or CBV data in addition to BOLD signals) will mitigate against the uncertainty inherent in BOLD signals. An important new direction for high field strength fMRI research will be to improve understanding of neurovascular and hemodynamic variability across heterogeneous subcortical structures, for instance across the thalamus, hippocampus, or adjacent component structures of the basal ganglia. Since fMRI methods can be applied in both human and animal models, these efforts will be best served by designing studies that are as far as possible analogous between species (i.e., in terms of stimulation paradigms, pulse sequences, anatomical focus), with animal models presenting an opportunity for more detailed investigation of key findings using additional, invasive techniques (as described above).

### Characterization of hemodynamic impulse response functions (HIRF)

**Figure 6:**
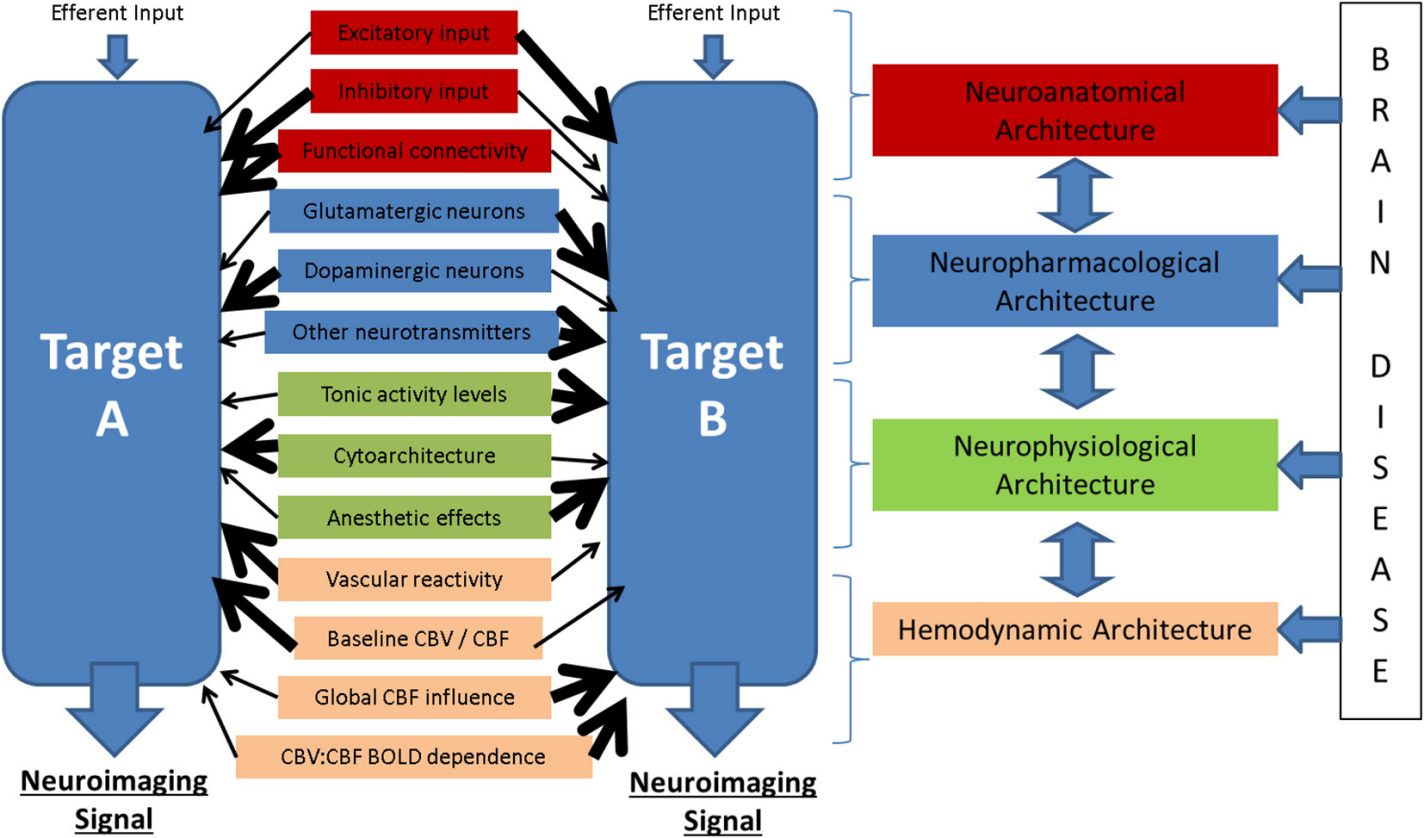
**Potential modulators of the neuronal-neuroimaging signal relationships illustrated in Figure 1**. When considering two neuroimaging targets, which may be: (1) different brain regions, (2) the same region in different subjects, (3) the same region in the same subject at different time points, or (4) the same region with before and after an experimental manipulation, it is important to consider the many possible differences between these targets that might affect the relationship between the measured hemodynamic and the inferred neuronal events.

The “hemodynamic impulse response function” (illustrated in Figure 2) is widely used as a canonical model in fMRI data analysis tools and as such a detailed understanding of this and how this is affected by brain region, health and disease status, pharmacological or physiological manipulations, is very important (Gitelman et al., 2003; Martindale et al., 2003; Handwerker et al., 2012). In essence, the HIRF refers to the hemodynamic response that results from a single neuronal event: by convolving this function with either measured neuronal activity or more commonly an estimate of the neuronal input function, an estimate of the neuroimaging signal change attributable to the stimulus is obtained. The HIRF is often approximated as a composite of two gamma functions which may be specified by a relatively small number of parameters. In fMRI analysis these parameters can either be fixed or allowed to vary within a predefined range in order to optimize the identification of responsive voxels. Although the general form of the temporal HIRF has been well-characterized and there is recent evidence that it is stable in the context of robust alterations in hemodynamic baseline parameters (Kennerley et al., 2012), in human studies as well as in animal experiments it has been shown that there are many factors that can influence the parameter values that specify the HIRF (Figure 6), as reviewed by Handwerker et al. (2012). A further complication is introduced by Hirano et al. (2011) who argue that a discrepancy between the BOLD and CBF HIRF that becomes apparent when moving from very brief to longer stimuli, is related to differential contributions of venous and arterial hemodynamic changes to the neuroimaging signals at different time points.

Even less well-understood is the spatial structure of the HIRF, yet specification of this is equally important in the context of spatiotemporal neuroimaging methods and the signal processing required. Within the cortex, the distinct neuronal and vascular architecture that is present in different layers is known to be a source of variability in hemodynamic responses, as described in the previous section. There have however been very few experimental studies investigating the spatial relationships between neuronal and hemodynamic signal changes across the cortical surface or in sub-cortical structures. One exception is Vazquez et al. (2013), who investigated the hemodynamic point-spread function using a multimodal stimulation and data-acquisition approach and showed a linear relationship between the spatial extent of neuronal and hemodynamic responses. One reason for a lack of research into the spatial correspondence of neuronal and hemodynamic changes that is required to inform development of a spatial HIRF is the availability of methods to simultaneously provide spatiotemporal readouts in both measurement modalities (e.g. see Baraghis et al., 2011). One solution is to investigate spatial hemodynamic changes in the context of neural systems with very well-known spatial response properties, such as the primary visual cortex (e.g. Aquino et al., 2014). In the context of optical imaging techniques it may also be possible to take advantage of rapid changes in light scattering that occur (due to cell swelling) when neurons are activated, or to separate optical signal components (e.g. from intrinsic metabolic and hemodynamic markers) using multichannel acquisition systems (Zhao et al., 2014b). In section Relating signals in time and space, we review other research into the temporal and spatial relationships between neuronal and hemodynamic signal changes which will also be important in the refinement of the canonical HIRF and how it is used.

Understanding the sources of HIRF variability could lead to improvements in neuroimaging experimental design, data analysis or interpretation. This understanding could also produce possibilities for differences in the HIRF to be exploited as biomarkers for the neuronal, vascular, or neurovascular perturbations of function associated with many brain diseases. Future goals could include the development a framework for neuroimaging data analysis that applies empirically derived constraints on a flexible HIRF according to (e.g.) brain region, subject age, health status, etc. Alternately, methods for obtaining subject or brain region specific HIRF estimates in human neuroimaging could be investigated (Kang et al., 2003; Handwerker et al., 2012) with the aim of developing a rapid scanning protocol which can help “tune-up” the canonical HIRF to be used in the analysis of the main study neuroimaging data.

### Investigations of neurovascular coupling mechanisms

A full review of insights into the biochemical or physiological mechanisms known to underpin neurovascular coupling is beyond the scope of this paper and there are a number of excellent recent reviews which address this issue directly (e.g. Attwell et al., 2010; Carmignoto and Gomez-Gonzalo, 2010; Cauli and Hamel, 2010; Hillman, 2014). In overview, combining the methodologies detailed in section Investigating neuroimaging signals using experimental animal models and Table 1 with pharmacological manipulations has been useful in elucidating, for instance through selective inhibition, key mechanisms, molecules, and mediators involved in neurovascular coupling. It is partly through this approach for instance that the roles of nitric oxide (Akgoren et al., 1994; Lindauer et al., 1999; Kitaura et al., 2007) and cyclooxygenases (Niwa et al., 2000, 2001; Lecrux et al., 2012) in neurovascular coupling have been elucidated, or the relationships between cerebral blood flow changes and oxidative metabolism have been probed (Leithner et al., 2010). This approach also enables the impact of therapeutic or other drugs upon neurovascular coupling or brain imaging signals to be investigated, either to explore these effects directly or to inform fMRI data interpretation in (for example) patient groups (Choi et al., 2006; Lindauer et al., 2010; Chin et al., 2011; Sander et al., 2013).

Most recently, possibilities for using optogenetic tools to help dissect the cellular-specific contribution to BOLD signals and neurovascular coupling have become available. Work using these methods is in its earliest stages. It has been applied to investigate the spatial correspondence of hemodynamic and neuronal responses to light-activation vs. sensory stimulation (Vazquez et al., 2013; Li et al., 2014) and in addition Lee et al. (2010) combined optogenetic stimulation with fMRI in mice and demonstrated a complex pattern of both positive and negative BOLD signal changes attributable to specific activation of cortical pyramidal cells (Lee et al., 2010; Urban et al., 2012). As in other areas adopting optogenetic approaches, there is a proliferation of new viral vectors and transgenic lines that allow targeting of specific components parts of the overall system (for example, astrocytes, Figueiredo et al., 2011) and as such it seems likely that these approaches will provide important insights into the contributions of elements of the neurovascular “circuit” to hemodynamic and fMRI signal changes. Whilst there are a finite number of cell types which contribute to neurovascular coupling, the number of pharmacological components of the key signaling pathways is much larger and by way of their interactions, substantially more complex. Combining new optogenetic tools and in particular inhibitory techniques (e.g. via halorhodopsin) with pharmacological manipulations will perhaps provide the most important insights, as this will allow interrogation of cell-specific biochemical pathways. It should also be noted that hemodynamic signal changes, and in particular when detected using fMRI, may be vulnerable to localized heating effects caused by laser power, as recently reported by Christie et al. (2012) and as such the use of optogenetic tools in neurovascular research will require careful validation.

A long standing debate in the literature which has recently become more prominent and which is likely to benefit from the development of optogenetic tools, concerns the relative contributions of neurons, astrocytes, and pericytes to neurovascular coupling. Whilst the release of neurotransmitters and vasoactive molecules from neurons is well established as a primary factor in initiating vascular responses, how to attribute the subsequent biochemical cascade resulting in locally increased blood flow to specific molecules and cell types remains uncertain. On the one hand a recent paper provided evidence for stimulus induced vasodilation occurring independently of the astrocyte-located mechanism thought to be critical for initiating the blood flow response (Nizar et al., 2013), yet conflicting results were subsequently reported by Lind et al. (2013). For an in-depth review of the complex role of astrocytes in neurovascular coupling, see Howarth (2014). Two very recent publications are likely to initiate new lines of enquiry in this field. Firstly Hall et al. (2014) outline a major and previously neglected role for pericytes in highly localized and dynamic control of blood flow in the brain and in a review Hillman (2014) suggests vascular endothelial cells may represent an additional overlooked mechanism in the regulation of brain blood flow and the generation of neuroimaging signals. The increasingly apparent complexity of neurovascular coupling may suggest a degree of physiological redundancy in the regulation of brain blood flow, an evolutionary consequence of the very limited oxygen available for sustaining the brains high metabolic demand should blood flow be perturbed. A finding by Leithner et al. (2010) that 30% of the hemodynamic response to evoked neuronal activity remains even when the major known biochemical pathways mediating neurovascular coupling are simultaneously inhibited by a cocktail of pharmacological agents, and that this reduction had little impact on neuronal activity, certainly seems indicative of system about which there is much to discover.

These uncertainties are important for the interpretation of neuroimaging signals in a number of ways. Knowing the biochemical or cellular substrates of hemodynamic signal changes, the relative contributions of direct neuron-vascular signaling pathways or signaling mediated via other cell types, will provide basic insights into the information contained within neuroimaging signals about (for example) neural computation or functional connectivity. Furthermore, variation in the contribution or functioning of these pathways between brain regions, disease states, age, and many other conditions could alter the provision of such information, and therefore have implications for how we interpret neuroimaging data in many situations.

In summary of this section, research into neurovascular coupling and the neurophysiological basis of neuroimaging signals is advancing beyond a broad-brush validation of neuroimaging signals as a marker for neuronal activity changes. It is now detailing a wider empirical landscape that will be needed for the interpretation of functional brain imaging signals acquired in increasingly diverse experimental and empirical contexts. The next sections highlight research in these areas that challenges simplistic interpretations of fMRI signals, suggesting that a more nuanced and contextually informed approach to signal analysis and data interpretation is indeed now required.

## Interpreting functional brain imaging signals as neuronal activity

In Figure 6 we summarize the factors which may influence what neuroimaging signals tell us about neuronal activity and which may differ between many conditions including brain regions, subjects, patient groups, and time-points. With respect to the interpretation of functional MRI signals, neuronal activity is usually broadly classified into two types: LFPs or spiking activity. Much research and discussion has focused upon the relative contributions of these two types of activity to BOLD signals and although it is generally recognized that neuroimaging signals correlate best to LFPs (Logothetis et al., 2001; Viswanathan and Freeman, 2007), spiking activity has also been shown to correlate closely in many (Logothetis et al., 2001; Jones et al., 2004), but not all contexts so far investigated (Caesar et al., 2003; Thomsen et al., 2004; Rauch et al., 2008). Another question that has been extensively investigated using experimental animals is whether brain hemodynamic responses are linearly, or non-linearly related to changes in specific aspects of neuronal activity. Research in which simultaneous neuronal and hemodynamic measures are made in cortex has shown that both linear and non-linear patterns of coupling are found which may be attributable to a range of factors (Jones et al., 2004, 2008; Sheth et al., 2004; Hewson-Stoate et al., 2005; Martin et al., 2006b; Hoffmeyer et al., 2007; Zhang et al., 2009; Liu et al., 2010; Magri et al., 2011). The issues of linearity and contributions of spiking or synaptic activity become more complex when the relative contributions of different neuronal input pathways (e.g. Enager et al., 2009) and neuron types to neurovascular coupling are considered. For example, it has been shown that both excitatory (glutamatergic) and inhibitory (GABAergic) neurons can evoke positive BOLD signals, whilst activity amongst inhibitory neurons alone can produce negative BOLD signals (Lauritzen et al., 2012).

It is important to note that much of the research that has been conducted to investigate the relative contributions of neuronal activity types to neuroimaging signals has focused almost exclusively on cerebral and to a lesser extent cerebellar cortical structures. Human neuroimaging on the other hand, is applied to study the whole brain and signals originating from subcortical structures are tacitly interpreted in the same way as cortical signals, despite the relative lack of empirical research to support such an approach. Indeed, the research that has been conducted suggests that neurovascular coupling is brain region dependent (Sloan et al., 2010; Devonshire et al., 2012), a finding that is perhaps not unsurprising given we know that brain structures differ substantially in their cytoarchitecture, vascular density, involvement of different neurons and neurotransmitter systems, and other factors (see Figure 6). We argue that as human neuroimaging continues to provide surrogate markers for activation across the whole brain, experimental animals studies will be needed to probe the regional heterogeneity of neurovascular coupling and the relative contributions of the various components of neuronal activity. Key questions to ask of specific brain structures include:

- What types of activity, in which neurons and under the influence of which efferent inputs are represented in fMRI signals?
- Are the “activation” thresholds for functionally meaningful neuronal activity (e.g. as determined through behavioral assay) and fMRI detectable hemodynamic changes equivalent?
- What are the baseline or resting state characteristics of neuronal activity, metabolism or hemodynamics upon which experimentally evoked changes are superposed?

### Relating signals in time and space

Detailed investigation of the spatiotemporal evolution of the hemodynamic response in experimental animal models has demonstrated that as it propagates through the capillary network and begins to include lager contributions from upstream or downstream changes in arterioles and venules respectively, the estimation of neuronal signal changes from hemodynamic proxies inevitably becomes more difficult (Hirano et al., 2011; Yu et al., 2012). For instance a recent study investigated the spatial correlation between neuronal activity and fMRI BOLD responses in the mouse somatosensory cortex using a combination of sensory stimulation and channelrhodopsin-mediated activation (Li et al., 2014). Focal activation of neurons using laser light produced neuronal responses that were tightly confined to the area of stimulation (~0.5 mm), whereas the hemodynamic responses to the same stimulus extended to a much larger area (>3 mm). Work by Vazquez et al. (2013) indicates a closer spatial correspondence of neuronal and hemodynamic changes where hemodynamics are measured using optical methods. This highlights how the ability of BOLD fMRI to spatially resolve neuronal activity changes is in part limited by biophysical factors such as large-vessel signal contributions to BOLD signals (see Kim and Ogawa, 2012, for a full review). In any case, it is evident that measuring the fine detail of the hemodynamic point-spread function, which may itself depend upon various factors (Figure 6), is important for mapping neuronal events.

Inverted hemodynamic responses corresponding to negative BOLD signal changes have also been studied in detail in animal models using optical imaging techniques. In both awake and anaesthetized rats, a center-surround response profile has been observed, where focal increases in cerebral blood flow, volume and oxygenation are accompanied by an inverted response annulus which itself appears to have a neuronal origin (Devor et al., 2007; Boorman et al., 2010; Martin et al., 2012). Although these “negative surround” responses are detectable using high field strength small animal fMRI (Kennerley et al., 2012), it is less likely that these small changes would be readily detectable in human fMRI, even though they may represent an important component of the overall response. The equivalent problem may also exist in the temporal domain. Our own work in awake rodents has revealed that the temporal hemodynamic responses function may have additional complexity which is masked by the commonplace us of anesthesia in *in vivo* research studies (see Discussion below and Martin et al., 2013). Specifically, we find evidence for a more dynamic, oscillatory hemodynamic response in cortex yet once again the limitations of (in this case temporal) resolution in typical human fMRI would make it unlikely that such changes would be detected (Martin et al., 2013). A recent theoretical work supports both of these empirical results, predicting the occurrence of both an adjacent region of negative BOLD response as well as temporal signal oscillations (Aquino et al., 2014).

Improvements in our understanding of the fine detail of the spatiotemporal hemodynamic response function would have two main benefits. Firstly, by enabling the derivation or estimation of a more accurate spatiotemporal HIRF (e.g. one that accounts for spatiotemporal hemodynamic oscillations, Aquino et al., 2014), we would be better able to estimate the magnitude and spatial extent of the underlying neuronal responses in standard fMRI studies. Secondly, as the spatial and temporal resolution of neuroimaging increases with greater availability of higher field strength magnets and other technological improvements, determining the finer grain detail of the spatiotemporal HIRF becomes more important to ensure parallel improvements in the accuracy of mapping neuronal activity in space and time using hemodynamic proxies. In both cases, it is clear that more research is needed to determine the spatiotemporal correspondence of hemodynamic responses to underlying neuronal activity, as well as how this changes across the brain.

### Quantification, baselines, and neuroenergetics

A major limiting factor for the interpretation of neuroimaging signals, especially those studies using BOLD fMRI, is the fact that BOLD signal changes are not quantitative. Signal changes are expressed as a percentage of baseline values which are themselves known to be influenced by a wide range of neurophysiological and general physiological factors. Because the BOLD signal is a product of cerebral blood flow, cerebral blood volume, and oxygen consumption (Figure 1), baseline changes affecting any or all of these properties will impact upon measured BOLD signals in ways that may not necessarily reflect commensurate changes in neuronal activity. For example, commonly ingested substances such as caffeine can alter BOLD baseline, in part through effects on oxidative metabolism and cerebral blood flow (Mulderink et al., 2002; Griffeth et al., 2011), as can aging, disease, or a range of pharmacological manipulations (e.g. D'esposito et al., 2003; Iannetti and Wise, 2007). In addition, Jones et al. (2008) showed that experimental alterations in baseline neuronal activity intended to emulate switching between different cortical arousal states affected neurovascular coupling in a rodent model. As discussed in section Limitations of animal models for neurovascular research, a major concern for the use of animal models in this field is the effects of anesthetic agents on baseline parameters.

One approach to tackling these difficulties has been to develop calibrated fMRI methods for use in humans which are able to provide quantitative measures of metabolic changes in response to task or stimulus conditions (see review by Hoge, 2012). This approach effectively reduces the uncertainty inherent in standard fMRI studies by providing a measurement that is more directly linked to neuronal activity changes, moving the research imperative from neurovascular coupling to neurometabolic coupling and neuroenergetics. Research in experimental animal models is making important contributions in this area and this endeavor is supported by recent evidence that the brain's energy budget, that is the attribution of brain energy consumption to different neuronal processes (Attwell and Laughlin, 2001), is preserved across mammalian species (Hyder et al., 2013). A full review of neurometabolic coupling and neuroenergetics is beyond the scope of the present paper (see Hyder and Rothman, 2012), however an important early finding from studies in rat was that energy use by neurons (oxidative glucose consumption) is linearly correlated to excitatory neuronal activity (glutamate release, Sibson et al., 1998). Understanding the relationship between oxidative glucose consumption and specific components of neuronal activity is therefore an important objective for interpreting quantitative neuroimaging signals. It is partly because of this and the knowledge that the metabolism of neurons and astrocytes is closely related (e.g. Pellerin and Magistretti, 1994; Bélanger et al., 2011), that research to understand the fine detail of neuron-astrocyte communication is so important for understanding functional brain imaging signals. Studies in which the activity of neurons and astrocytes can be differentiated and/or specifically manipulated, for example using optogenetic approaches combined with 2PLSM will be significant in this regard (Li et al., 2013a) and combination of these approaches with metabolic readouts, for instance using molecular oxygen sensors (Lecoq et al., 2011) or more macroscopic techniques (e.g. see Devor et al., 2012) will advance understanding considerably.

## Impact of disease and modulatory neurotransmitters upon the interpretation of functional brain imaging signals

Functional brain imaging is increasingly being applied to investigate brain function in clinical populations (e.g. Diamond et al., 2007; Karmonik et al., 2010; O'brien et al., 2010; Sundermann et al., 2014), it is important to be able to conduct a detailed exploration of the impact of brain diseases upon neuroimaging signals and their relationship to neuronal activity in animal models. This includes the use of both transgenic lines and pharmacologically or surgically induced disease states (Sanganahalli et al., 2013; Serres et al., 2014). Such investigations may become more important as neurovascular breakdown becomes increasingly implicated in a range of disease conditions (Zlokovic, 2010, 2011), suggesting that the empirical basis of assumptions concerning neuroimaging signal interpretation which have been established through research primarily in healthy animals, may not apply. If neuroimaging techniques are to be used to investigate the effects of therapeutic drugs on brain function biomarkers, it will also be important for example to delineate (neuro)vascular and neuronal effects of these drugs. For example, a study in human Alzheimer's patients suggested that acetycholinesterase inhibitors produced alterations in cortical vascular response to stimuli that were independent of changes in the underlying neuronal response (Rosengarten et al., 2009). In addition, the impact of other perturbations of normal brain function including normal aging (D'esposito et al., 2003; Rosengarten et al., 2003) and the ingestion of substances known to alter neurovascular and/or hemodynamic function such as caffeine (Pelligrino et al., 2010; Diukova et al., 2012) or alcohol (Luchtmann et al., 2013) upon these signals can also be explored in depth (Meno et al., 2005; Diukova et al., 2012).

Because functional brain imaging signals are the final consequence of neuronal, neurometabolic and hemodynamic events, they are vulnerable to disease-related perturbations operating at a number of levels. Although alterations in neuronal function as a result of pathology are in general likely to be reflected within altered neuroimaging signals, a key question however is to what extent these signal changes also reflect alterations in the normal translation of neuronal events to hemodynamic changes? The use of neuroimaging data to estimate differences in neural signals within subjects, between groups or experimental conditions, or across experimental animals, tacitly assumes preserved or at least non-systematically altered neurovascular coupling. There is however accumulating evidence that many brain diseases feature altered neurovascular coupling and that in estimating neuronal signals using hemodynamic based imaging techniques, these effects must be taken into account.

### Neuroimaging in neurodegenerative disease

There is a growing consensus that changes in the function of the neurovascular unit are a critical component of brain disease development and progression (Benarroch, 2007; Zacchigna et al., 2008; Iadecola, 2010; Grammas, 2011; Zlokovic, 2011). In the case of neurodegenerative disease, alterations in neurovascular function have been detected at early stages, preceding the onset of clinical indicators (Bookheimer et al., 2000; Ruitenberg et al., 2005; Knopman and Roberts, 2010; Sheline et al., 2010; Zlokovic, 2011) and it is now well established that cerebrovascular dysfunction is a major risk factor for many brain diseases (Girouard and Iadecola, 2006; Iadecola, 2010; Toledo et al., 2013). A number of studies have identified changes in vascular reactivity, hemodynamic responses or neurovascular coupling in animal models of neurodegenerative disease (Rancillac et al., 2012; Sanganahalli et al., 2013). In relation to this, neuroinflammation has been identified as a key neurodegenerative disease process involving neurovascular unit disruption. Whilst acute neuroinflammatory responses may have a neuroprotective function, chronic inflammatory responses within the central nervous system are associated with neuronal damage and may not only “fans the flames” of many CNS disorders (Frank-Cannon et al., 2009), but may precede and even play a causal role in these diseases (Hauss-Wegrzyniak et al., 1998; Qin et al., 2007; Gao et al., 2011; Cunningham, 2013).

Work carried out in specific animal models of disease has also suggested disease-related alterations in neurovascular function that may affect our ability to interpret fMRI signal changes. In the area of neurodegeneration for instance, a widely used transgenic model is one in which the amyloid-beta precursor protein (APP) is over-expressed, leading to a toxic accumulation of amyloid-beta protein. Such models recapitulate many, but not all (for a review see Balducci and Forloni, 2011) of the major features of human AD including an age-dependent neurovascular impairment and vascular dysfunction (Lecrux and Hamel, 2011). Work in these mice shows, amongst other things, impaired hemodynamic responses to neuronal activation, altered resting cerebral blood flow and cerebrovascular autoregulation and impaired metabolic activity (Iadecola et al., 1999; Niwa et al., 2002; Nicolakakis et al., 2008). Additionally, ApoE4 mice model the known association in humans between AD (and cerebral amyloid angiopathy, CAA) and possession of the apolipoprotein E ε 4 allele (ApoE4). Expression of ApoE4 has been linked to higher risk of AD with earlier onset (Blacker et al., 1997) and is believed to be related to dysfunctional clearance of amyloid-beta from the brain (Castellano et al., 2011; Hawkes et al., 2012). It has been suggested that the *APOE*ε4 allele may alter neurovascular function through amyloid-beta exerting a time- and concentration-dependent toxic effect on rat microvascular endothelial cells (Folin et al., 2006). In addition, fMRI readouts of brain hemodynamic signals have found that in human ApoE4 carriers, cerebral blood flow, task-related brain activity, and functional connectivity appear altered several decades prior to any clinical indicators of dementia (Filippini et al., 2009, 2011).

In addition to Alzheimer's disease and other neurodegenerative conditions, alterations in neurovascular coupling have also been reported in a number of other diseases and pathological states (Girouard and Iadecola, 2006; Hamilton et al., 2010) including stroke and ischemia (Lin et al., 2011; Baker et al., 2013; Jackman and Iadecola, 2014), hypertension and hypotension, spreading depression (Hamzei et al., 2003; Nagaoka et al., 2006; Del Zoppo, 2010; Ayata, 2013; Fordsmann et al., 2013) as well as in normal aging (D'esposito et al., 2003). There is also accumulating evidence for longer term effects of systemic health challenges upon brain microcirculatory regulation and neurovascular coupling. For example, a recent study demonstrated impairment of neurovascular coupling in animals fed a high fat diet over a period of several weeks (Li et al., 2013b) and systemic infection has been show to produce alterations in cerebrovascular function (Puntener et al., 2012) and the shape of hemodynamic responses (Couch et al., 2013). Finally, many brain diseases involve alterations in the function of specific neurotransmitter systems, and we refer the reader to the next section for an exploration of the possible effects of such alterations.

Overall, there is substantial scope for disease conditions and potentially the health status of individuals more generally to impact upon how we should interpret functional neuroimaging data. By understanding more specifically how these alterations impact upon neurovascular function and the relationships between neuronal and hemodynamic changes, it may be possible to either optimize data analysis strategies to account for these effects or build consideration of these changes into our discussions of neuroimaging findings. Lastly, we speculate that early alterations in neurovascular function associated with many diseases may be detectable through concurrent measurement of neuronal and hemodynamic activity in the brain. As such, these neurovascular changes may provide for novel disease biomarkers, measureable using multimodal techniques that are now becoming available for use in humans. We return to this in the last section of this review.

### Neurotransmitter and neuropharmacological modulations of neurovascular coupling

Although the established view of the role of specific neurotransmitters in neurovascular coupling processes emphasize predominantly glutamate and GABA (e.g. Logothetis, 2008), recent evidence has emerged suggesting that other neurotransmitters may also play a role. The serotonin, noradrenaline, dopamine, and acetylcholine systems each feature neurons that project widely throughout cortical and subcortical structures, in addition to forming key elements of certain specific structure-structure connections. A fundamental challenge for the use of hemodynamic imaging methods in contexts where function in these neurotransmitter systems (and relevant structures) arises from the fact that they are vasoactive (Choi et al., 2006; Hamel, 2006; Martin and Sibson, 2008; Jenkins, 2012; Shih et al., 2012b; Toussay et al., 2013), and therefore able to elicit hemodynamic effects which may not be directly related to their effects upon neuronal activity. We will focus here on the emerging evidence for effects of dopamine and serotonin neurotransmission upon neurovascular coupling and the interpretation of neuroimaging signals, although similar lines of evidence exist for modulation of neuroimaging signals by other neurotransmitters including acetylcholine (Hamel, 2006; Rosengarten et al., 2006; Kocharyan et al., 2008) and noradrenaline (Toussay et al., 2013).

The neurotransmitter dopamine (DA) and the functional anatomy of the dopaminergic system has been intensively studied for many years. This is due in part to the known involvement of this neurotransmitter in a range of cognitive, affective and motor functions in healthy brain as well as a wide range of diseases including Parkinson's disease, schizophrenia, drug addiction, ADHD, and pain disorders to name a few. Unsurprisingly, there is a rapid proliferation of non-invasive brain imaging studies of both healthy and disease-related brain processes that are associated with alterations in DA function, for instance, fMRI studies of Parkinson's disease (Hacker et al., 2012), risk-taking behavior (Kohno et al., 2013), schizophrenia (Yoon et al., 2013), reward prediction error (Chowdhury et al., 2013). This literature, which addresses pharmacological, disease- or task-related, or genetic alterations in DA function, rests on the assumption that differences in fMRI signals between conditions or subjects are attributable to effects of the altered DA function on neuronal activity. We suggest that sufficient data to support this assumption does not yet exist. Although arguments have been made that fMRI signals may provide an adequate biomarker of DA release (Knutson and Gibbs, 2007), this does not address the issue of how to interpret stimulus/task evoked changes in neuronal activity in the context of altered DA function. In addition, we know that the long established vasoactive properties of dopamine are capable of producing complex effects upon the relationship between DA signaling, neurovascular coupling and fMRI responses in subcortical structures (Devonshire et al., 2004; Choi et al., 2006; Shih et al., 2009; Jenkins, 2012; Mandeville et al., 2013).

Due in part to its non-quantitative nature, alterations in baseline parameters including metabolism, cerebral blood flow, and oxygenation renders fMRI signal interpretation vulnerable to the effects of such changes upon both evoked responses and resting-state measurements. As it has been shown that the magnitude of stimulus-evoked BOLD responses is dependent upon such baseline parameters (Shulman et al., 2007; Lu et al., 2008), the effects of changes in dopaminergic neurotransmission upon local baseline conditions such as cerebral blood flow and brain metabolism is an important potential confound. In relation to this, direct regulation of brain microvasculature tone by dopamine has been demonstrated (Krimer et al., 1998; Choi et al., 2006; Kowianski et al., 2013) and such vasomotor regulation has the capacity to modulate measured fMRI signal changes (Tian et al., 2010), with these effects occurring independently of neurogenic neurovascular coupling and this potentially confounding interpretation of fMRI data. For instance, Arthurs et al. (2004) measured the effects of the selective dopamine D2 receptor antagonist sulpiride on electrophysiological and fMRI responses in human subjects and using path analysis estimated that 84% of the drug effect on fMRI responses to stimulation occurred via direct effects upon hemodynamics, rather than neuronal responses themselves (Arthurs et al., 2004).

fMRI is also widely used to investigate the role of 5-HT in normal function, disease processes, and therapeutic drug effects in a wide range of human and animal models (Hariri et al., 2002; Hariri and Weinberger, 2003; Del-Ben et al., 2005, 2008; Mckie et al., 2005; Stark et al., 2006, 2008; Rao et al., 2007; Tanaka et al., 2007; Graeff and Del-Ben, 2008; Munafo et al., 2008). Within human fMRI studies, responses in structures receiving serotonergic input appear to be profoundly influenced by synaptic 5-HT, with up to 42% of fMRI response variability attributable to availability of the 5-HT transporter (Rhodes et al., 2007). Windischberger et al. (2010) report a powerful modulation of BOLD responses to stimuli by selective serotonin reuptake inhibitors (SSRIs) that was specific to regions receiving dense serotonergic projections. However, many research studies point to a complex role for serotonin in neurovascular coupling (Cauli et al., 2004; Hamel, 2006), vascular reactivity (Bonvento et al., 1997) and for the interpretation of BOLD fMRI signals in terms of spiking or synaptic activity (Rauch et al., 2008). Additionally, the well-established influence of ascending serotonergic projections on the cerebral vasculature and microvascular tone (Toda and Fujita, 1973; Dieguez et al., 1981; Cohen et al., 1996, 1999) provide strong potential for modulation of vascular compliance and therefore BOLD signal dynamics (Behzadi and Liu, 2005; Boas et al., 2008). This severely complicates (a) the interpretation of neuroimaging signals from structures receiving serotonergic input, and (b) comparisons of task-evoked responses between conditions involving altered 5-HT function.

## Limitations of animal models for neurovascular research

Although work in animal models is essential to advance the capabilities of hemodynamic neuroimaging methods to estimate neuronal activity in humans, there are a number of important limitations associated with their use. A major concern is the use of anesthesia in the vast majority of animal research carried out in this field. Anesthesia is used for two main purposes. Firstly, as many of the techniques used to study neuronal, neurovascular and hemodynamic processes are invasive, anesthesia is necessary in order to prevent suffering associated with (for instance) the placement of recording electrodes. Secondly, anesthesia is often used in order to prevent movement of the animal during data collection. It is possible to conduct both imaging (e.g. using fMRI or optical techniques) and electrophysiological experiments in un-anaesthetized animals and indeed this approach has been taken by a number of laboratories (see below). For small animal imaging in particular therefore, anesthesia is frequently used chiefly to prevent movement, avoiding the need for restraint and/or length animal training protocols. Because all general anesthetics have at least moderate effects upon normal physiological regulation (for example blood pressure, blood oxygenation, thermoregulation) it is usually necessary to perform additional invasive procedures (even if the imaging technique is itself non-invasive) in order to enable normal physiology to restored, monitored and maintained.

Using anesthesia in animal research models has important consequences for translation of findings from *in vivo* experimental research to the human neuroimaging arena (where subjects are rarely imaged under anesthesia). Previous research in our laboratory and elsewhere has indicated that anesthetic agents disrupt neurovascular coupling in a number of ways (Lahti et al., 1999; Nakao et al., 2001; Brevard et al., 2003; Sicard et al., 2003; Martin et al., 2006b, 2012; Luo et al., 2007; Tsurugizawa et al., 2010; Fukuda et al., 2013), including via alterations in baseline hemodynamic parameters (Shulman et al., 1999; Hyder et al., 2002). A recent study comparing the effects of four different anesthetics on BOLD fMRI responses in mice found that response differences could largely be explained by differing systemic effects of the stimuli attributable to the different anesthetic conditions (Schroeter et al., 2014).

A relatively consistent finding is that anesthetics significantly delay the hemodynamic response function (Martin et al., 2006b; Huttunen et al., 2008; Franceschini et al., 2010) and although a recent study comparing fMRI BOLD responses in awake and anesthetized marmosets found the opposite effect (more protracted responses in awake animals, Liu et al., 2013), the authors suggest this was in turn the result of the much larger responses observed and consequent increases in venous drainage time. A further complication is that different anesthetic agents disrupt neurovascular coupling in different ways. This may be particularly problematic for pharmacological neuroimaging studies. For example Du et al. (2009) demonstrated anesthetic-dependent effects upon hemodynamic changes induced by cocaine including alterations in the coupling between different hemodynamic measures. A study investigating the role of nitric oxide in neurovascular coupling found differences between awake and anesthetized conditions that were in turn attributable to the deleterious effects of the anesthetic agent upon the magnitude of stimulus-evoked cerebral blood flow changes (Nakao et al., 2001). For a more in depth discussion of the effects of anesthesia in studies of neurovascular coupling the reader is directed to a recent review paper (Masamoto and Kanno, 2012).

Much human fMRI engages subjects in complex cognitive tasks which involve activity within, and communication between, networked structures. Models of many relevant cognitive and affective processes such as learning, memory and attention have been established in awake behaving animals, as well as capabilities to study disease-relevant alterations in these functions. An additional problem with the use of anesthesia in animal models therefore is that it limits the possibilities for investigating neurovascular function, and therefore improving our understanding neuroimaging signals, in the context of these “higher-order” brain functions. Although a number of laboratories have developed procedures for conducting fMRI or other hemodynamic measurement procedures in awake animals (Martin et al., 2002, 2013; Brevard et al., 2003; Sicard et al., 2003; Chin et al., 2011; Desai et al., 2011; Brydges et al., 2013; Liu et al., 2013; Pisauro et al., 2013; Takuwa et al., 2013), and a few studies also report on the neuronal signals underlying the hemodynamic and/or fMRI responses (Lipton et al., 2006; Martin et al., 2006b; Goense and Logothetis, 2008; Sirotin and Das, 2009; Desai et al., 2011; Liu et al., 2013), this work continues to focus almost exclusively on cortical structures. In addition, the effects of stress, especially where animals are restrained, must be carefully taken into account as this is likely to have a range of general physiological and neurophysiological effects. This is particularly the case were animal models of neuropsychiatric illness or related drug treatments are being studied. It will also be a challenge to establish animal models for cognitive or behavioral neuroscience in the context of the apparatus generally required for investigation of neuroimaging signals and neurovascular coupling (as outlined in section Investigating neuroimaging signals using experimental animal models and Table 1), where restraint is required. To reduce restraint stress and allow more sophisticated experimental designs, methods for fixing experimental animals with respect to data acquisition apparatus but permitting engagement in cognitive tasks have recently been reported (e.g. Dombeck et al., 2007), and one study reports a method whereby rats voluntarily engage with head-restraint apparatus to allow functional brain imaging (using 2PLSM).

## Future research challenges and possibilities

We suggest that a key contribution of future research studies in animal models will be the investigation of neuronal-hemodynamic-neuroimaging signal relationships in subcortical structures, using both awake and anesthetized animals. To optimize the translation of neurovascular coupling data from animal studies to improving neural signal estimation in human fMRI, it will also be important to study neurovascular coupling in the context of information processing or in behavioral paradigms that more closely reflect research designs used in human subjects. A major challenge in this respect will be the use of restraint in most of the current awake animal studies. A small number of laboratories have already successfully investigated affective or cognitive processes using fMRI in awake rats (e.g. see review by Ferris, 2014; also Brydges et al., 2013; Zhao et al., 2014a) and the combination of these approaches with either concurrent measurement of neuronal data, or careful synthesis of new hemodynamic data with existing data regarding underlying neuronal activity, will provide important insights. Methods have also been recently developed for use in rodents that utilize virtual reality technology in order to provide head-fixed animals with a pseudo-environment with which they can interact (Harvey et al., 2009; Scott et al., 2013). Adaptations of these techniques may further provide key insights into the neuronal events that fMRI signals report on whilst the brain is engaged in relatively complex tasks.

Technological developments are increasing the possibilities to investigate the neuronal basis of hemodynamic neuroimaging signals directly in humans. For example, combining EEG with fMRI (EEG-fMRI) is an approach that has primarily been deployed to marry the relatively high spatial resolution of fMRI with the high temporal resolution of EEG for cognitive neuroscience research purposes (Huster et al., 2012; Laufs, 2012). This approach may also provide insights into neurovascular coupling in human subjects (Wan et al., 2006; Diukova et al., 2012; Huster et al., 2012; Mayhew et al., 2013; Mullinger et al., 2013) although the spatial and temporal mismatch of the measurements made by each technique will pose a number of challenges: the spatial and temporal domains from which data are sampled are very different. Other approaches include simultaneous near infra-red spectroscopy (NIRS) and EEG (Moosmann et al., 2003), combined MEG and NIRS (e.g. Mackert et al., 2004) or diffuse optical imaging combined with MEG (Ou et al., 2009). Most recently, Fabiani et al. (2014) report on a combined optical spectroscopy, event-related potential and fMRI study of change in neurovascular function in normal aging.

## Summary

As non-invasive brain imaging techniques are used to address an increasingly diverse range of questions relevant to both brain function and dysfunction, it will become more important that our understanding of the neurophysiological basis of these signals is specific to the brain structure, disease context, pharmacological, or task-related effects under investigation. Fortunately a wide range of experimental tools, including combinations of methods that optimize our ability to probe neuroimaging and neuronal signal relationships, are now available in experimental animals. It will be important that these approaches continue to develop to enable neurovascular coupling to be probed in contexts established in behavioral neuroscience. Simultaneously, technical advances in human research studies that allow neurovascular coupling to be probed directly will help detail a more comprehensive empirical framework for estimating neural signals in the human brain from hemodynamic proxies.

### Conflict of interest statement

The author declares that the research was conducted in the absence of any commercial or financial relationships that could be construed as a potential conflict of interest.
